# Adding glimepiride to current insulin therapy increases high-molecular weight adiponectin levels to improve glycemic control in poorly controlled type 2 diabetes

**DOI:** 10.1186/1758-5996-6-41

**Published:** 2014-03-20

**Authors:** Chun-Jun Li, Jing-Yun Zhang, De-Min Yu, Qiu-Mei Zhang

**Affiliations:** 1 2011 Collaborative Innovation Center of Tianjin for Medical Epigenetics, Key Laboratory of Hormone and Development (Ministry of Health), Metabolic Disease Hospital, Tianjin 300070, China; 2 Tianjin Institute of Endocrinology, Tianjin Medical University, Tianjin 300070, China

**Keywords:** Glimepiride, Insulin therapy, HMW adiponectin, Type 2 diabetes

## Abstract

**Background:**

To observe the efficacy and safety of adding glimepiride to established insulin therapy in poorly controlled type 2 diabetes (T2D) and to assess the relationship of changes in the serum high-molecular weight (HMW) adiponectin levels and glycemic control after glimepiride treatment.

**Methods:**

Fifty-six subjects with poorly controlled insulin-treated T2D were randomly assigned to either the glimepiride-added group (the group A, n = 29) or the insulin-increasing group (the group B, n = 27) while continuing current insulin-based therapy. Glycosylated hemoglobin (HbA1c) value, daily insulin dose, body weight, waist circumference, plasma lipid concentration, serum HMW adiponectin level and the number of hypoglycemic events were evaluated before and after treatment.

**Results:**

At the end of study, insulin doses were significantly reduced, and the mean HbA1c, fasting blood glucose (FBG) and 2-hour postprandial blood glucose (P2BG) were improved greater in the group A compared with the group B. The serum HMW adiponectin levels were significantly increased in the group A compared with the group B. Most importantly, we found that changes in HbA1c were inversely correlated with changes in serum HMW adiponectin in the group A (r = −0.452, p = 0.02).

**Conclusions:**

Adding glimepiride to current insulin treatment led to better improvement in glycemic control with a significant smaller daily insulin dose, and the increases in the serum HMW adiponectin levels may directly contribute to improvement glycemic control.

## Introduction

Tight glycemic control with either intensive insulin therapy or sulfonylurea has been associated with weight gain in patients with type 2 diabetes (T2D) [1,2]. Achieving glycemic control is a critical metabolic goal because hyperglycemia contributes to the progression of T2D by adversely affecting both β-cell function and insulin sensitivity [3]. In clinical practice, achieving sustained glycemic lowering over time is an important aspect of therapy that is not achieved in many patients treated with large dosage of exogenous insulin combination with non-sulfonylurea drugs [4]. In addition, new treatment paradigm, aimed at reducing insulin resistance may represent a more effective treatment to decrease insulin dose. Unfortunately, most of the insulin sensitizers such as thiazolidinediones (TZDs) are associated with chronic heart failure [5]. Increasing the insulin doses or changing the insulin regimen to multiple injections of insulin could improve glycemic control. However, both endogenous hyperinsulinaemia and exogenous insulin could increase the risk of atherosclerosis and cancer [6], meanwhile some physicians have concerns about hypoglycemia and weight gain with intensive insulin treatment [7].

Glimepiride, a third-generation sulfonylurea (SU), exerts its effects mainly by stimulating insulin secretion but has also been shown to have extrapancreatic effect such as improvement insulin resistance [8]. Low level of adipnectin was associated with insulin resistance, obesity, and T2D [9]. Adiponectin is specifically and abundantly expressed in adipose tissue and directly sensitizes the body to insulin, which exists as three forms: a trimer of low molecular weight, a hexamer of medium molecular weight and a larger multimeric high molecular weight (HMW) form [10]. Glimepiride is reported to increase adiponectin gene expression in adipocytes [11]. And several studies have also demonstrated that glimepiride may increase in insulin sensitivity associated with increased serum adiponectinemia [12-14]. The HMW adiponectin has been the active form of the hormone and has relevant role in enhancing insulin sensitivity and in protecting against diabetes [15]. Recently, several studies have observed pioglitazone therapy significantly improving glycemic control and markedly increasing serum HMW adiponectin levels in T2D [16,17]. However, it is still unclear whether glimepiride treatment in T2D patients could increase serum HMW adiponectin level to improve glycemic control. And limited studies have reported that combination therapy with glimepiride and insulin could improve glycemic control and reduce insulin requirements [18,19], but the mechanisms are not clear. Therefore, the present study was conducted to analyze the relationship between the degree of lowering HbA1c and serum HMW adiponectin levels and provide predictors of which patients would benefit from addition glimepiride in poorly controlled T2D subjects with insulin therapy.

## Materials and methods

This study was undertaken in the out-patient setting of the Metabolic Disease Hospital of Tianjin Medical University. Subjects eligible for the study met the following criteria: T2D was defined by Chinese Diabetes Association and HbA1c exceeding 8% treated by large dosage of insulin (daily insulin dose more than 40 units) for at least 6 months. Subjects were excluded if they had hepatic injury (serum alanine or aspartate aminotransferase 2.5 of more times the upper-normal range), or congestive heart failure (NYHA Class III or IV) or renal damage (serum creatinine above 2.0 mg/dl), and those already receiving sulfonylureas or insulin sensitizers such as TZDs within 6 months prior to the recruitment. Eligible subjects were explained the goals and risk of the study and gave their written informed consent before beginning the study. The study protocol was approved by the Tianjin Medical University Ethics Committee Review Board and was conducted in accordance with the Declaration of Helsinki and Good Clinical Practice guidelines. Fifty-six subjects with T2D were randomly assigned into either the glimepiride-added group (the group A, n = 29) or the insulin-increasing group (the group B, n = 27) while continuing based therapy.

HbA1c value, daily insulin dose, body weight, and the number of hypoglycemic events were recorded at weeks 0, 12 and 24. Plasma lipid concentrations, serum C peptide and HMW adiponectin concentration were measured at weeks 0 and 24. The glycemic control target was defined as fasting blood glucose (FBG) ≤ 7.0 mmol/L and 2-hour postprandial blood glucose (P2BG) ≤ 10 mmol/L. In the group A, glimepiride (Amaryl, Sanofi aventis) was initiated at the minimum dosage 1 mg once daily and then titrated up to 4 mg daily until the glycemic control target. In the group B, insulin doses were increased to reach the glycemic control target. Hypoglycemic episodes and adverse events (AEs) were recorded throughout the study. Hypoglycemia was determined by the number of blood glucose readings that were below 3.9 mmol/L, or occurrences of definite hypoglycemic symptoms. Hypoglycemia was considered severe when the event required third party assistance. Adverse events were classified as serious if they resulted in death, life-threatening experiences, hospitalization, or persistant of significant disability or incapacity.

In addition, we have divided the group A subjects into two sub-groups, according to the degree of HbA1c lowering (Responder, greater than 0.5% HbA1c lowering, Non-responder, less than 0.5% HbA1c lowering). Plasma total cholesterol, high-density lipoprotein (HDL) cholesterol, and triglyceride concentrations were assessed using standard enzymatic methods. HbA1c was assayed using high-performance liquid chromatography. The HMW adiponectin was measured with commercial ELISA Kit (R&D Systems, USA).

### Statistical analysis

Normally distributed data are expressed as mean ± standard deviation and non-normally distributed data median or as numbers and percentages. Non-normally distributed data were logtransformed for use with parametric statistics. Unpaired t test was used to compare the differences in clinical characteristics between groups at baseline and after treatment assessed for significance using for the discrete or continuous data and the chi-square test for frequency distributions. The changes in HbA1c, FBG and 2 h-BG over time (at baseline and at the other 2 visits) were studied using the repeated measurements ANOVA with treatment as grouping factor. Paired t tests were used to compare within-group changes. And unpaired t tests were also used to compare baseline variables between Responders and Non-responders in subjects treated with glimepiride-added. Linear regressions were performed to determine relationships between changes in serum HMW adiponectin levels and changes in HbA1c. The statistical analyses were performed using SPSS windows version 18.0, and p value < 0.05 was considered to be statistical significance.

## Results

A total of 56 subjects with poorly controlled insulin-treated T2D were recruited and completed the trial. At baseline, the two groups did not differ regarding anthropometric data, duration of diabetes and insulin treatment, BMI, waist circumference, body weight, HbA1c value, FBG, P2BG, plasma lipid concentration, daily insulin dose, frequency of insulin injections, combined anti-diabetic agents, C-peptide concentration and HMW adiponectin levels. Overall, the subjects had a long duration of diabetes and poor glycemic control with large dosage of insulin. 75% of the subjects had abdominal obesity defined by Chinese Diabetes Association (Male waist circumference ≥ 90 cm, Female waist circumference ≥ 85 cm). A similar portion of subjects in the two groups were suffering from diabetic complications such as nephropathy, retinopathy and cardiovascular disease (Table 1).

**Table 1:** Baseline characteristics of subjects in the 2 groups

**Clinical characteristics**	**The group A (n = 29)**	**The group B (n = 27)**
Age (years)	56.8 ± 12.3	56.3 ± 12.4
Sex (Male/Female)	15/14	13/14
Body weight (kg)	66.2 ± 16.3	67.2 ± 15.9
BMI (kg/m^2^)	25.2 ± 3.6	25.4 ± 3.7
Waist Circumference (cm)	93.55 ± 10.5	93.46 ± 10.4
duration of diabetes (years)	15.6 ± 5.7	15.4 ± 6.2
Hypertention (+/−)	22/7	21/6
Retinopathy(NDR/SDR/PDR)	2/17/10	2/16/9
Nephropathy(Normo-/micro-/macroalbuminuria)	6/14/9	5/14/8
CVD(−/+)	5/24	5/22
α-glucosidase inhibitors(-/ +)	2/27	1/26
Metformin (−/ +)	13/16	14/13
Duration of insulin treatment (years)	9.6 ± 3.4	9.4 ± 3.5
Total insulin dose (unit/day)	68.6 ± 14.6	66.8 ± 13.9
Frequency of insulin injection (2-/3−/−4 times /d)	17/8/4	16/7/4
HbA1c (%)	9.3 ± 1.5	9.4 ± 1.4
FBG (mmol/L)	11.4 ± 2.4	11.3 ± 2.6
P2BG (mmol/L)	16.8 ± 4.4	17.0 ± 4.5
Triglyceride (mmol/L)	3.26 ± 1.14	3.28 ± 1.16
Total Cholesterol (mmol/L)	6.23 ± 1.41	6.19 ± 1.51
HDL-Cholesterol (mmol/L)	1.04 ± 0.23	1.12 ± 0.21
LDL-Cholesterol (mmol/L)	4.13 ± 1.01	4.20 ± 1.02
Serum C peptide (ng/ml)	1.06 ± 0.68	1.06 ± 0.70
HMW adiponectin (μg/ml)	3.12 ± 1.56	3.22 ± 1.54

Normally distributed data expressed as mean ± standard deviation and non-normally distributed data expressed as median or as numbers and percentages. Non-normally distributed data were log-transformed for use with parametric statistics. BMI: body mass index; NDR: no diabetic retinopathy; SDR: simple diabetic retinopathy; PDR: proliferative diabetic retinopathy; CVD: cardiovascular disease; HbA1c: Glycosylated hemoglobin; FBG: fasting blood glucose; P2BG: 2-hour postprandial blood glucose; LDL: low-density lipoprotein; HMW adiponectin: high-molecular weight adiponectin.

During the study, mean values of HbA1c, FBG and P2BG were significantly reduced in both groups (Table 2). Repeated measurements of ANOVA on decreases in HbA1c, FBG and P2BG over the time were significant different between groups and greater in the group A (p < 0.01). The primary efficacy endpoint, the reductions in HbA1c of 2.0% from baseline in the group A was significant greater than reductions of 1.6% in the group B (p < 0.01). The target HbA1c level 7.0% achieved by 15 subjects (52%) in the group A was larger than 8 subjects (30%) in the group B (p < 0.05). The required insulin doses were reduced by 53% (from 68.6 ± 14.6 to 32.3 ± 14.9 unit/d) in the group A, while it was increased by 29% (from 66.8 ± 13.9 to 86.2 ± 18.9 unit/d) in the group B, the difference is significant. Body weight was increased significantly during the course of the study in the group B (from 67.2 ± 15.9 kg to 69.8 ± 16.5 kg, p <0.05); however, there was no significant change in body weight in the group A (from 66.2 ± 16.3 to 66.9 ± 16.6 kg, p > 0.05).

**Table 2:** Assessment of treatments for efficacy in glycemic control in the 2 groups

**Variables**	**0 week**	**12 weeks**	**24 weeks**
HbA1c (%)			
The group A	9.3 ± 1.5	8.2 ± 1.2*	7.3 ± 1.3**
The group B	9.4 ± 1.4	8.6 ± 1.3*	7.9 ± 1.3*^#^
FBG (mmol/L)			
The group A	11.4 ± 2.4	7.6 ± 1.8**	6.9 ± 1.3**
The group B	11.3 ± 2.6	8.2 ± 2.1**	7.7 ± 1.6**^#^
P2BG (mmol/L)			
The group A	16.8 ± 4.4	11.2 ± 2.3**	8.6 ± 2.1**
The group B	17.0 ± 4.5	12.4 ± 2.3**	10.8 ± 2.2**^#^
Insulin dose (unit/day)			
The group A	68.6 ± 14.6	44.8 ± 15.9**	32.3 ± 14.9**
The group B	66.8 ± 13.9	79.3 ± 14.8*	86.2 ± 18.9**^#^^#^
Body Weight (kg)			
The group A	66.2 ± 16.3	66.4 ± 16.5	66.9 ± 16.6
The group B	67.2 ± 15.9	68.3 ± 16.0	69.8 ± 16.5**^#^

Data expressed mean ± SD; HbA1c: Glycosylated hemoglobin; FBG: fasting blood glucose; P2BG: 2-hour postprandial blood glucose; *p < 0.05 vs. baseline, **p < 0.01 vs. baseline. ^#^p < 0.05 group A vs. group B, ^##^p < 0.01 group A vs. group B.

There were no significant changes in plasma triglyceride, total cholesterol, LDL-cholesterol, HDL-cholesterol and plasma C peptide in both groups. Serum HMW adiponectin levels were markedly increased from (3.12 ± 1.56) μg/ml to (5.86 ± 1.62) μg/ml in the group A, while no significant changes in the group B (Table 3). Changes in HbA1c were inversely associated with changes in serum HMW adiponectin in the group A (r = −0.452, p = 0.02). Reinforcing this, when we stratified patients into subgroups according to the degree of HbA1c lowering ≥ 0.5% or 0.5%: the responder group (n = 23) and the non-responder group (n = 6), there were significant greater increases in the HMW adiponectin concentrations in the responder group compared with the non-responder group. In addition, there were higher levels of body weight, waist circumference, HbA1c, FBG, total insulin doses and lower HMW adiponectin concentrations in the responder group compared with the non-responder group at baseline (Table 4). However, a weak but significant linear correlation was found between the baseline HbA1c values and changes in HbA1c after treatment (r = −0.38, p = 0.04), and no significant correlation was found between baseline serum HMW adiponectin and changes in HbA1c (r = 0.29, p = 0.15).

**Table 3:** Changes in outcome parameters after 24 weeks treatment

**Variable**	**The group A (n = 29)**		**The group B (n = 27)**	
	**Baseline**	**24 weeks**	**Baseline**	**24 weeks**
Triglyceride (mmol/L)	3.26 ± 1.14	3.16 ± 1.12	3.28 ± 1.16	3.24 ± 1.15
Total Cholesterol (mmol/L)	6.23 ± 1.41	6.18 ± 1.72	6.19 ± 1.51	6.17 ± 1.68
HDL-Cholesterol (mmol/L)	1.04 ± 0.23	1.14 ± 0.38	1.12 ± 0.21	1.13 ± 0.24
LDL-Cholesterol (mmol/L)	4.13 ± 1.01	3.67 ± 1.12	4.20 ± 1.02	3.81 ± 1.20
Serum C peptide (ng/ml)	1.06 ± 0.68	1.18 ± 0.66	1.06 ± 0.70	1.10 ± 0.69
HMW adiponectin (ug/ml)	3.12 ± 1.56	5.86 ± 1.62*	3.22 ± 1.54	3.24 ± 1.53^#^^#^

Data expressed mean ± SD; LDL: low-density lipoprotein; HMW: high-molecular weight; *p < 0.05 difference compared with baseline, ^##^p < 0.01 compared absolute changes between the group A and group B following 24 weeks glimepiride treatment.

**Table 4:** Changes in the responders and non- responder following 24 weeks treatment

**Variables**	**Responder group (n = 23)**		**Non-responder group (n = 6)**	
	**Baseline**	**24 weeks**	**Baseline**	**24 weeks**
Body weight (kg)	66.8 ± 16.3	67.3 ± 17.4	62.4 ± 14.8*	64.3 ± 15.9
Waist Circumference (cm)	97.6 ± 12.2	98.0 ± 12.1	84.6 ± 85**	85.9 ± 8.4
Total insulin dose (units/day)	71.3 ± 14.9	32.1 ± 14.0	59.8 ± 12.9 **	54.5 ± 14.7^##^
HbA1c (%)	9.5 ± 1.2	7.8 ± 1.1	8.6 ± 1.3*	8.4 ± 1.1^##^
FBG (mmol/L)	12.0 ± 2.7	7.4 ± 1.4	9.8 ± 2.1*	9.2 ± 1.5^##^
P2BG (mmol/L)	16.7 ± 4.8	9.8 ± 2.4	16.3 ± 4.2	15.8 ± 4.2^##^
HMW adiponectin (ug/ml)	2.89 ± 1.54	5.79 ± 1.58	4.74 ± 1.46**	5.11 ± 1.52^##^

Normally distributed data expressed as mean ± standard deviation and non-normally distributed data expressed as median or as numbers and percentages. Non-normally distributed data were logtransformed for use with parametric statistics. HbA1c: Glycosylated hemoglobin; FBG: fasting blood glucose; P2BG: 2-hour postprandial blood glucose; HMW: high-molecular weight; Data are mean ± SD. *p < 0.05 and **P <0.01 for responder vs. non-responder at baseline; ^#^p<0.05 and ^##^p<0.01 for absolute changes in responder vs. non-responder following 24 weeks glimepiride treatment.

During the 24-week observation period, hypoglycemic episodes were significantly lower in the group A than in the group B (p < 0.05); Moreover, significantly fewer subjects in the group A experienced at least one hypoglycemic episode. Despite the occurrence of hypoglycemic episodes in the two groups, no episode was classified as severe, requiring assistance. Moreover, no AE was recorded during the study.

## Discussion

In the present study, subjects with poorly controlled T2D achieved significant mean reductions in the HbA1c, FBG and P2BG with glimepiride-added treatment compared with increasing insulin doses. A significant higher percentage of subjects (52%) in the group A had an HbA1c goal of ≤ 7% at the end of study compared with (30%) in the group B, reflecting the greater mean reduction in the HbA1c observed in the group A. Furthermore, adding glimepiride also remarkably decreased insulin doses and offered clear advantages in terms of a reduction in the incidence of hypoglycemia and no weight gain. The most interesting findings reported here were the significant correlations between HbA1c lowering and increases in serum HMW adiponectin levels following 24-week glimepiride treatment (r = −0.452, p = 0.02). We also found that serum HMW adiponectin levels increased particularly more in patients who got more than 0.5% reductions in HbA1c compared with patients who got less than 0.5%, suggesting the possible role of increases in serum HMW adiponectin levels on the glimepiride-induced glycemic control.

In practice, a significant number of subjects with T2D cannot achieve tight glycemic control despite treatment with large dosage insulin combination with non-sulfonylurea drugs over time. Most of physicians are reluctant to continue to increase insulin doses or to carry out intensive insulin treatment due to the side effects associated with insulin-induced hypoglycemia and weight gain. Combinations of insulin with second-generation sulfonylurea derivatives, such as glibenclamide and gliclazide, have been found to offer significant improvement in glycemic control with a significantly smaller daily insulin dose [20]. SU use with insulin works because of the higher levels of insulin in the portal circulation with SU, compared to subcutaneous insulin, lowers hepatic glucose output and therefore fasting insulin better. Although many SU agents have been administered successfully with insulin, only glimepiride has been approved by the United States Food and Drug Adiministration for combination therapy. Moreover, only glimepiride has been shown not to block the beneficial effect of myocardial ischemic preconditioning that glyburide and glipizide have [21]. Furthermore, a large cohort study recently provided the clinical evidence of a trend toward an increased overall mortality risk with glyburide or glipizide versus glimepiride in those with documented cardiovascular disease [22]. Therefore, we preferred to add glimepiride to the current insulin therapy in the present study. Several clinical studies have supported that addition of glimepiride in the subjects with poorly controlled insulin-treated type 2 diabetes could improve glycemic control and reduce insulin requirements [18,19]. However, the underlined mechanisms are still not clear.

Insulin resistance has been the main reason for gradual worsening of glycemic control in obese T2D subjects. Obese T2D has become a big problem in Asian subjects, more susceptible to suffer from abdominal obesity compared with European and American subjects [23]. In our study, 75% subjects with poorly controlled glycemia had abdominal obesity with large dosage of insulin. In obese subjects, circulating adiponectin concentrations inversely correlate with visceral fat area, but not with BMI, and subcutaneous fat area [24]. Reduction of visceral fat increases circulating adiponectin levels in the general population and obese individuals [8]. Several clinical trials have shown that glimepiride could increase serum adiponectin levels in T2D [12,13,25]. The HMW adiponectin is the main active form of the hormone and has relevant role in enhancing insulin sensitivity and protecting against diabetes [15]. For the first time, the present study demonstrated that glimepiride combination with insulin therapy could markedly increase serum HMW adiponectin levels in T2D subjects. We also found a significant negative correlation between changes of HbA1c and increases in HMW adiponectin levels following 24-week glimepiride treatment, suggesting that the greater reduction in HbA1c is associated with the greater increases in HMW adiponectin levels. This suggests that the reduced glycemic effect of glimepiride may be mediated through up-regulation and increased secretion of HMW adiponectin by adipocytes [25]. Moreover, several studies supported that insulin seems to suppress expression and secretion of adiponectin both in vitro [26] and in vivo studies [27], suggesting that the great reductions in insulin dose by glimepiride treatment may conversely increase plasma HMW adiponectin concentration. Nagasaka et al. [13] suggest that the increase in adiponectinemia by the glimepiride treatment could be, in part, due to an effect of glycemic control. Therefore, the mechanisms of the increased adiponectinemia by glimepiride may be complex and multifactorial.

Hypoglycemia is the most important barrier associated with anti-diabetic treatment. During the 24-week observation period, adding glimepiride treatment is associated with lower hypoglycemic episodes compared with increasing insulin doses to the insulin-based therapy. Moreover, 67% of the subjects treated with increasing insulin doses experienced at least one episode of hypoglycaemia, while only 28% of the subjects with adding glimepiride experienced at least one episode of hypogycaemia. Glimepiride is widely used second-generation sulfonylurea purportedly has lower risk of hypoglycaemia and weight gain relative to other medications in the same class [28,29]. The risk of hypoglycemia may be dose-dependent; the relationship between dose and glycemic efficacy of sulfonylurea agents was not linear over the therapeutic dose range. In a dose-range finding study with glimepiride, a substantial reduction in HbA1c was observed with 1 mg daily, whereas nearly full efficacy was reached with 4- mg/day dose with an additional efficacy obtained by escalation through doses of 8-mg/day [30]. Overall, clinical AE and drug-related AEs were reported more frequently with higher doses glimepiride.

Weight gain is another problem associated with insulin treatment. As reported by the UKPDS, subjects with T2D gained about 4 kg body weight after 10 years of insulin treatment [2]. As expected, in the study, the mean body weight was slightly increased in the group B after 24 weeks insulin administration. The gain in body weight associated with insulin and sulfonylurea agents is an undesirable side effect in subjects with T2D. In the study, we surprisingly found no significant weight gain with glimepiride-added to insulin therapy, which maybe benefit from remarkably decreased insulin doses. Obesity correlates with diabetogenic, atherogenic, pro-thrombolic, and pro-inflammatory metabolic CVDs, which increase the risk of atherosclerotic CVD [23]. Even a slight increase in body weight appears harmful, as it is associated with increased mortality and serious comorbidities, such as hypertension, hyperlipidemia, and CVD. Therefore, the subjects in the group B seem to have greater risk of severe metabolic abnormalities.

## Conclusions

In summary, we firstly showed in this study that adding glimepiride led to better improvement in glycemic control with a significant smaller daily insulin dose, especially in the abdominal obese T2D subjects with lower HMW adiponectin level before treatment. Most importantly, there was a significant negative correlation between the increase in the HMW adiponectin levels and changes in the HbA1c following glimepiride treatment. And the HMW adiponectin level increased particularly more in responder subjects compared with non-responder subjects, suggesting that increases in the serum HMW adiponectin level may directly contribute to improvement glycemic control. A limitation of the study was the relatively small sample size and would require further research to prove our results.

## Competing interests

The authors declare that they have no competing interests.

## Authors’ contributions

DMY and QMZ conceived the study, analyzed data and wrote the manuscript. CJL and JYZ acquired and analyzed data, and wrote the manuscript. All authors read and approved the final manuscript.
